# An Account of the Results of a Series of Experiments to Ascertain the Influence of Certain Substances on the Secretion of the Urine, with a View to the Elucidation of the Etiology of Diabetes

**Published:** 1820-01

**Authors:** 

**Affiliations:** Halle.


FOR THE LONDON MEDICAL AND PHYSICAL JOURNAL.

### *An Account of the Results of a Series of Experiments to ascertain the Influence of certain Substances on the Secretion of the Urine, with a View to the Elucidation of the Etiology of Diabetes.* By Dr. Krimer, of Halle.

1°. THE long-continued use of the farina of rye renders dogs dull and indolent: a discharge of a puriform, mucous, fluid takes place from their eyes; and uric acid is diminished in their urine, in proportion as the quantity of albumen and mucus is augmented: but the action of the voltaic pile removes those morbid phenomena, and produces a prompt return to health.

2°. The use of French or black wheat, (*fagopyrum*, cl. apetales, § 3, Tournefort,) produces no obvious effect, neither on dogs nor on rabbits: on the contrary, the decoction of common wheat (*triticum*) increases the quantity of the urine, renders it alkaline, and gives it a faint, sickly, odour: the animal also becomes very voracious.

3°. Rabbits nourished with ale fall sick, and perish with all the signs of gastritis and enteritis.

4°. Rice, merely boiled, produces the same effects in dogs as the rye-meal, but less intense in degree. The uric acid totally disappears; the urea is also in part deficient; whilst the proportion of albumen is considerably increased. The action of the voltaic pile soon restores them to health.

5°. When dogs or rabbits are made to swallow diabetic urine, diabetic sugar is found in their urine at the end of twenty-four hours. The quantity of this substance afterwards increases as long as the diabetic urine is given to the animal, and even for some days after this regimen has ceased.

6°. Animals who often take diabetic urine do not contract the disease, and remain in good health, excepting the change in the urine already noticed.

7°. A rabbit, however, died after having several times taken that urine, without the cause of its death being discernible. The body presented nearly the same appearances as that of the subjects of diabetes. The vessels of the kidneys were very much dilated, and readily permeable to injected fluids.

8°. As soon as the diabetic sugar appears in the urine of animals, the urea either partially or totally disappears.

9°. If a little syrup prepared with diabetic urine be injected into the veins, or into the stomach, of an animal, sugar is found for some time in the arterial and venous blood, as well as in the urine. If, on the contrary, immediately after the injection of the syrup into the veins, the animal be submitted to the action of a galvanic pile of moderate power, no sugar is found either in the blood or in the urine; but the vapour exhaled from the lungs, during expiration, appears to contain it.

10°. After injecting an aqueous solution of common sugar into the veins of an animal, no trace of this substance is found either in the urine or blood; but it is evident in the product of pulmonary perspiration.

11°. If all the urinary passages of an animal be forcibly electrified, ardent thirst is soon manifest; and a more abundant secretion of thick, heavy, urine, charged with a large quantity of albumen and the colouring matter of the blood, ensues.

12°. After the use of the carbonate of potash or of soda, the urine of animals becomes clearer, and somewhat more heavy than before; the uric acid almost totally disappears, but the quantity of urea is augmented.

13°. When a certain quantity of carbonate of ammonia is introduced into the stomach of an animal, the urine, from having been acid, becomes ammoniacal: it contains much more urea, and but little uric acid; and it quickly passes to putrefaction.

14°. The urine of animals which have taken carbonate of magnesia, contains a considerable quantity of carbonate and phosphate of magnesia. But if, a little before the administration of this substance, some phosphate of ammonia be injected into the stomach, the urine voided soon afterwards contains a considerable quantity of ammoniaco-magnesian phosphate, under the form of a yellowish-white flocculent substance.

15°. The long-continued use of carbonate of lime, produces an abundant precipitate of phosphate of lime in the urine; but if, at the same time with that, carbonate of potash be also given, the calculous deposition from the urine becomes still more considerable, and the animal soon dies. After death, traces of inflammation of the kidneys are found, and the urinary passages become coated with a layer of phosphate and carbonate of lime.

16°. Sulphate of lime does not produce any obvious change in the functions of a dog, although administered for a considerable length of time.

17°. When bile is administered to a dog, that fluid is found in the urine, and the urea is increased.

18°. When bile is injected into the veins of an animal, the tunica sclerotica becomes coloured yellow; the arterial, as well as venous, blood contains that fluid: but, as soon as the bile appears in the urine, the urea disappears; and the animal becomes dull, and his activity depressed, when the experiment is frequently repeated.

19°. The same phenomena are produced by biliary calculi dissolved in water, and injected into the stomach or veins of an animal.

It was found from experiments, undertaken with other views, that the secretion of the urine is in some way connected with the influence of the fifth pair of nerves; and the diminution of the action of these nerves is followed by an increase of the quantity of albumen, of mucus, and of the colouring matter of the blood; so that it seems probable that the production of sugar in diabetes depends on an analogous state of the nerves of the par vagum.

Some cases of diabetes which lately occurred to the observation of M. Demours, and of which he read an account to the *Cercle Medicale*, seem to be worthy of being connected with the foregoing observations; as they seem to show the intimate connexion of the origin of that disorder with derangement of the functions of the nervous system, and especially with depression of nervous power. The conjecture of Dr. Krimer respecting the relation of the functions of the par vagum to the production of the malady in question, is also favoured by them, in the debility of the functions of the stomach in connexion with signs of nervous asthenia.

A man, thirty-two years of age, of small stature, of a healthy constitution, and moderately-full habit of body, was exposed to a storm in the middle of a forest, and could not find any place of refuge. He arrived home two hours afterwards, his clothes wetted through, and suffering much from cold. A few weeks had hardly elapsed, when he perceived that his stomach ceased to perform its functions as well as ordinarily, that he became thin in person, lost his strength, and especially that his sight became very weak. He then came to Paris, and consulted M. Demours. No other disorder of the eyes was discernible than a slight dilatation of the pupils, and these were only conjectured to be larger than ordinarily; the alternate contractile and dilatative motions of the iris took place nearly the same as in the healthy state: yet the patient could not see sufficiently well to be able to read; and, indeed, he could hardly walk about without a guide. With the partial amaurosis and the other symptoms of debility already mentioned, the patient complained of all the symptoms of diabetes mellitus. This malady had been present four months, and became manifest about the same time as the weakness of sight. The patient having lived near one of his own iron-foundries, in the environs of Saarbruck, had not been able to have the advice of a well-informed physician, and had been treated in a very vague and otherwise improper manner. M. Thenard examined his urine, and found it to present the ordinary composition of that found in diabetes. M. de la Motte, M. Dupuytren, and M. Demours, met in consultation on the 8th of November, 1817. It was ordered that the enormous quantity of drink which the patient had been taking, should be replaced by only a single pint of chalybeate water daily, which he was to take by spoonfuls, and only to appease his thirst; that bread, and all other vegetable nutriment, should be suppressed; and that he should live on animal food alone. The patient, being obliged to attend to some particular business on the 9th, was to have commenced this treatment on the 10th; but he died suddenly about noon on the foregoing day. On opening his body, there was no evident alteration of structure in the optic nerves, nor in the brain, nor in the kidneys, nor in any of the abdominal viscera.

General de B——, forty-five years of age, of a constitution naturally robust, and of what is called the sanguineous temperament, came, in October 1818, to Paris, to consult M. Demours. He was afflicted with chronic inflammation of the margins of the eye-lids; the most evident and immediate cause of which seemed to be, a diminution of the natural quantity of the lachrymal fluid. He complained of dryness of his mouth; general lassitude, especially on waking in the morning; and of a gradual, though not very remarkable, decline of his muscular powers. He suffered continual thirst, which drink did not relieve; and he was habitually costive in his bowels. He had lost flesh to a very considerable extent. The sound of his voice was altered, from the dryness of the mouth and pharynx. The urea had disappeared from his urine, which had become sweet to the taste, and, by chemical analysis, was found to be similar to that generally voided by persons having diabetes: it was clear, nearly colourless, and inodorous, and was in quantity about double that of the fluids drunken; that is, from sixteen to twenty pints in the twenty-four hours. His diet had been legumens, with but a small portion of animal food. He had been in the habit of drinking very hot tea, night and morning, according to the custom of his country (Holland); and at length he had resided for a long time at Ypres, in Belgium, where he had made use of water of very ill quality. The weakness of the patient was not so great but that he was able to walk nearly two miles. M. Demours commenced by directing him some tonic medicines: his diet was also altered; the only drink he was allowed was a small quantity of wine; his food was changed to beef, ham, and game. On the third day, the urine had acquired a light-yellow colour, and the sweet taste was less sensible. All the morbid symptoms diminished rapidly; the inflammation of the eye-lids disappeared, on the natural secretion of the lachrymal fluid being established, in proportion as the quantity of urine was diminished. The functions generally acquired their ordinary vigour; and, at the end of three months from the commencement of the treatment, General B—— had entirely recovered his health. G.

*Paris; November* 16, 1819.

